# Antibiotic-Resistant Nontuberculosis Mycobacteria Infection after Liposuction and Fat Grafting and Full Thickness Cervical Burns after Renuvion Skin Tightening

**DOI:** 10.1093/asjof/ojaf018.015

**Published:** 2025-05-13

**Authors:** Colton Riley, Sarvam TerKonda, Olivia Ho

**Affiliations:** Mayo Clinic Florida, Jacksonville, FL; Mayo Clinic Florida, Jacksonville, FL; Mayo Clinic Florida, Jacksonville, FL

## Abstract

**Goals/Purpose:**

To address the complex problems arising from contemporary aesthetic procedures, specifically focusing on the occurrence of antibiotic-resistant non-tuberculosis mycobacterium (NTM) infections following autologous fat grafting and the development of full-thickness burns due to the use of Renuvion for skin tightening. The study underscores the critical need for stringent aseptic techniques, updated sterilization practices, and thorough understanding of new cosmetic technologies to prevent severe complications.

**Methods/Technique:**

A 45-year-old otherwise healthy female underwent autologous fat grafting to the face and neck tightening with Renuvion in 2023 at an outside aesthetic clinic to address premature facial and cervical ageing. Post-operatively, her course was complicated by multiple midface abscesses from fat grafting and third-degree burns to the neck from Renuvion. She initially sought treatment at a local community hospital, where aspiration was performed which revealed a Mycobacterium fortuitum infection. Despite debridement and antibiotics for the abscesses and xenograft application for her cervical burns, her symptoms persisted, prompting presentation to our tertiary care center.

On arrival to our facility, the patient exhibited multiple dermal and subcutaneous abscesses in the premaxillary soft tissues. Broad-spectrum antibiotics were initiated, and she underwent surgical incision and drainage. Cultures identified a multiple resistant Mycobacterium fortuitum complex. Following infectious disease consultation, she commenced a 10-month course of IV antibiotic therapy involving three drugs. Despite this, in 2024, she experienced on-going facial drainage, necessitating further debridement and antibiotic therapy.

**Results/Complications:**

The patient’s condition was complicated by the discovery of a multiply resistant Mycobacterium fortuitum complex. The infection's resistance to standard antibiotics required an extended and rigorous 10-month treatment regimen involving a combination of macrolide, aminoglycoside, and other antibiotics as well as surgical debridement. Despite aggressive treatment, persistent facial drainage necessitated further surgical intervention. The chronic and recurring nature of the infection significantly increased morbidity and highlighted the need for a multidisciplinary approach to optimize patient outcomes. Additionally, the use of Renuvion led to full-thickness burns, a complication not previously reported in the literature per extensive review.

**Conclusion:**

This case report and literature review highlights the increasing complexities and potential complications associated with modern aesthetic procedures, specifically antibiotic resistant NTM infections following autologous fat grafting and full thickness burns in the setting of liposuction and Renuvion skin tightening. It underscores the necessity for meticulous aseptic technique, thorough knowledge of equipment, and adherence to modern sterilization protocols to mitigate the risk of infection. Additionally, the development of thermal injuries with Renuvion emphasizes the need for increased reporting to enable proper training and caution with its application. This case report is limited by the inability to definitively identify the source or etiology of the mycobacterium infection and the specific method that led to the full thickness burn, which highlights the need for further investigation and reporting in these areas. This case report is intended to stimulate discussion, investing, and reporting to ultimately improve safety standards in cosmetic surgery and stresses the importance for patients to seek out board-certified and qualified plastic surgeons, as their expertise and adherence to the highest safety standards can significantly reduce the risk of complications such as these.

